# Editorial: Understanding the mechanism of dynamic cell communications: a much needed angle for cell motility and barrier functions

**DOI:** 10.3389/fmolb.2025.1663453

**Published:** 2025-07-25

**Authors:** Virag Vas, Zhen Xu, Pasquale Cervero

**Affiliations:** ^1^Institute of Molecular Life Sciences, HUN-REN Research Centre for Natural Sciences, Budapest, Hungary; ^2^Department of Neurosurgery, McGovern Medical School, The University of Texas Health Science Center at Houston, Houston, TX, United States; ^3^Institute for Medical Microbiology, Virology and Hygiene, University Medical Center Eppendorf, Hamburg, Germany

**Keywords:** cell motility, signaling, cancer biology, protein-protein interaction, interactome analysis

Intercellular communication is a fundamental aspect of cell biology, enabling cells to exchange messages with their surroundings and coordinate behaviours adaptively. This process is crucial for maintaining tissue homeostasis; therefore, it is not surprising that the disruptions in cell interactions can lead to various pathological conditions, such as cancers. Despite significant advancements, our understanding of the dynamic mechanisms underlying protein interactions during cell signaling, particularly in the context of tumor cell motility, remains incomplete. Addressing these gaps is essential for developing more effective diagnostic and therapeutic strategies, and this Research Topic sheds novel light on the functional consequences and the translational potential of the mechanisms of dynamic cell communication.

This editorial summarizes five significant research articles published in *Frontiers in Molecular Bioscience*s during 2024–2025, each contributing unique insights into diverse biological processes governed by signaling networks under both normal and disease states. These studies collectively demonstrated the interdisciplinary nature of modern molecular biology research, spanning reproductive endocrinology, oncology, neurobiology, through protein biochemistry, therapeutic targets, and diagnostic approaches.

Tilajka et al. contributed an insightful bioinformatic study dealing with the invadosome marker protein Tks4 (Tyrosine Kinase Substrate with Four SH3 domains) and its interaction network as potential biomarkers for colon cancer. Recognizing that Tks4 scaffold protein (encoded by SH3PXD2B gene) has established roles in invadopodia/motility and epithelial-to-mesenchymal transition (EMT) regulation, but remains understudied in colon cancer, the authors analyzed the expression of Tks4 and six associated partner molecules (CD2AP, GRB2, WASL, SRC, CTTN, CAPZA1) in colon tumor vs normal tissues. This study demonstrates that the co-expression patterns of these seven biomarker candidates provide improved diagnostic accuracy in distinguishing tumor from normal samples compared to the individual gene expression levels. Furthermore, the variable importance analysis identified four core genes (WASL, GRB2, SRC, and Tks4) within the interactome that maintain comparable diagnostic performance, suggesting the potential clinical utility of this reduced biomarker panel for colon cancer detection.

In their contribution, Spinelli et al. highlighted the critical role of another cellular adhesion, survival/proliferation and invasion mediating signaling pathway: the calpain/calpastatin proteolytic system in patient-derived glioblastoma stem cells (GSCs), revealing unique expression patterns for each GSC culture. They identify that hcast 3–25, a Type III calpastatin variant devoid of inhibitory units, potentially affects stem cell state and promotes a more differentiated, less aggressive phenotype of glioblastoma. Transfection experiments reveal that hcast 3–25 effectively associates with calpains and supports digestion of selected calpain targets. These findings potentially illuminate new therapeutic approaches for glioblastoma treatment by targeting stem cell functions.

The third study by Köper et al. provides novel insights into filopodia and membrane dynamics regulated by the protein known as Plasticity-Related Gene 5 (PRG5). This lipid-phosphate phosphatase is involved in the signal transduction organizing spine-like structures. The study employs advanced imaging techniques, particularly Fluorescence Lifetime Imaging (FLIM) to quantify Förster Resonance Energy Transfer (FRET), enabling visualization and quantification of PRG5 multimers at the plasma membrane in living cells. The results demonstrate that PRG5 forms multimers in cells, with distinct localization at the distal tip in neuronal spine-like structures as opposed to non-neuronal filopodia. This work represents the first evidence for PRG5 multimerization at specific cellular locations, suggesting functional roles in extracellular matrix interactions and membrane protrusion stability. The findings contribute significantly to the understanding of synaptic plasticity mechanisms and potential therapeutic targets for neurological disorders.

Despite the diverse systems applied in this Research Topic, these studies share common themes, each emphasizes how molecular complexes within protein networks determine cell behaviour. In the fourth research paper, Ibrahem et al. explored bioactive peptides derived from β-lactoglobulin (BLG) and their impact on apoptosis/angiogenesis networks in cancer cells. The authors hydrolyse bovine BLG with trypsin and find that a 60-minute hydrolysate is the richest in low-molecular-weight peptides with antioxidant activity. Mass spectrometry identifies 162 peptides, many of which are hydrophobic and cationic. The hydrophobic nature of the BLG hydrolysates might make this fraction prone to interact with cancer cell membranes, enabling their disruption and leading to cell death. Importantly, the BLG hydrolysate also exerts anticancer and anti-angiogenic effects *in vitro*: significantly increases apoptosis (via caspase-9 activation) while downregulating VEGFR-2 to inhibit angiogenesis. These findings suggest that specific BLG-derived peptides might serve as natural therapeutic agents. The authors propose isolating the most potent individual peptides for an upcoming study to enhance antitumor efficacy.

In summary, these studies jointly demonstrate that cellular phenotype is governed by complex intracellular and extracellular molecular interplays, which are dynamically connected through molecular-scale communications. The fifth study uncovered a novel long noncoding RNA (lncRNA) mediated regulatory interplay in polycystic ovary syndrome (PCOS). Yan et al. established a mechanistic model based on experimental results, in which the lncRNA called SNHG12 binds to the non-histone chromosome structural protein HMGB1, preventing HMGB1 from activating PTEN transcription, thereby promoting glycolysis. In line with this, reduced SNHG12 impairs glycolysis, increases granulosa cell apoptosis, and contributes to follicular dysplasia. Furthermore, the overexpression of SNHG12 lncRNA alleviated PCOS symptoms in the presented mouse model. This work confirms the crucial role of cellular metabolism in PCOS pathology and adds SNHG12/HMGB1/PTEN axis to the growing list of lncRNA-regulated pathways. Importantly, it identifies SNHG12 as both a biomarker and a potential therapeutic target. From a broader perspective, it exemplifies how noncoding RNAs can influence metabolic signaling, highlighting the importance of exploring RNA-targeted therapies for endocrine disorders.

In conclusion, the studies in this Research Topic collectively demonstrate that the integration of biochemical assays, imaging techniques, and computational analyses are multifaceted approaches that will guide future research in understanding the mechanisms of dynamic cell communications.

